# Does adiposity mediate the relationship between socioeconomic position and non-allergic asthma in childhood?

**DOI:** 10.1136/jech-2017-209722

**Published:** 2018-01-24

**Authors:** Kate Marie Lewis, Hynek Pikhart, Joana Morrison

**Affiliations:** 1 Population, Policy and Practice, UCL Great Ormond Street Institute of Child Health, London, UK; 2 Research Department of Epidemiology and Public Health, University College London, London, UK

**Keywords:** BMI, bodyweight, cohort study, mediator, non-atopic asthma, obesity, socioeconomic position, waist circumference

## Abstract

**Background:**

Despite its high prevalence, early onset and chronic nature, the causes of asthma are not clearly established. The present study examined a plausible but untested relationship in the development of non-allergic asthma; an asthma phenotype closely linked to deprivation and other preventable risk factors. Our aim was to determine the mediating role of adiposity in the relationship between socioeconomic position in infancy and non-allergic asthma emergence in mid-childhood.

**Methods:**

To estimate the causal indirect effect of adiposity we applied the parametric g-computational procedure to 6203 singleton children from the UK Millennium Cohort Study. Adiposity was measured at age 7 by body mass index, waist circumference and waist circumference-to-height ratio. Children who developed non-allergic asthma between the age of 7 and 14 were compared with children without allergies or allergic asthma at these ages.

**Results:**

We found no evidence to suggest that adiposity is a mediator in the relationship between socioeconomic position and the development of non-allergic asthma in mid-childhood. After adjustment for risk factors, the direct effect of socioeconomic position remained; children in the lowest tertile of socioeconomic position had a 43% (OR 1.43, 95% CI 1.38 to 1.49) greater odds of developing non-allergic asthma compared with the highest tertile.

**Conclusions:**

Adiposity at age 7 does not mediate the relationship between socioeconomic position and non-allergic asthma. The results suggest that improving socioeconomic conditions and promoting healthy weight are both important in reducing the development of non-allergic asthma in early to mid-childhood.

## Introduction

Asthma is a chronic respiratory condition that develops in 15% of the English population by their early teens.^1^ Annual National Health Service asthma care costs are estimated at almost £1 billion, which is the value before taking into account the cost of the numerous comorbidities and disadvantage associated with asthma across the life course.^2 3^ Despite the evident burden of asthma, efforts to prevent the development of the condition have been limited due, in part, to a restricted understanding of the preventable risk factors for asthma.^4^ Attempts to address the socioeconomic determinants of this chronic condition have had mixed results. Strina *et al*^5^ and others^4^ argue that differentiation between asthma with and without accompanying allergy is necessary due to the different causal mechanisms involved.^6^ Allergic asthma is linked to common allergens and comorbidities such as hay fever and eczema, whereas non-allergic asthma is not.^7^ This research will focus on non-allergic asthma, which, when examined independently from allergic asthma, has been consistently linked with lower socioeconomic circumstances and related risk factors.^4–7^ Epidemiological research from England previously identified asthma without allergy in 7.6% and asthma with allergy in 11.9% of children 7–8 years of age.^4^

One important factor associated with both socioeconomic position and non-allergic asthma is adiposity.^5 8 9^ Markedly, pathways to both overweight/obesity and asthma embed at an early age.^10 11^ Individuals with both asthma and obesity typically have worse health outcomes including poorer asthma control, worsened respiratory symptoms and lower self-reported quality of life.^9 12^ Although independent links between socioeconomic position, excessive bodyweight and non-allergic asthma have been made,^13^ the potential association between the three factors has not yet, to the authors’ knowledge, been investigated. In addition, research has typically focused on body mass index (BMI), when other indicators may determine adiposity more precisely in children.^8 14 15^ The aim of this study was to determine whether adiposity is a mediator in the relationship between socioeconomic position in early childhood and the development of non-allergic asthma in mid-childhood. Previous research on similar topics used cross-sectional design, adult only samples and non-differentiation of asthma phenotypes.^4 6 8 14 16 17^ This current study will use longitudinal cohort data to help improve understanding of mid-childhood adiposity as a target for intervention to reduce the inequity of this burdensome respiratory condition.

## Data and methods

### Conceptual framework

To establish the extent to which the socioeconomic effect on non-allergic asthma is mediated through adiposity, this study adopted a counterfactual-based approach to mediation analysis as described by VanderWeele.^18^ This framework decomposes the total effects of the exposure (in this case socioeconomic position) on the outcome (non-allergic asthma) into natural indirect and direct effects. The natural indirect effect captures the exposure-outcome effect that is due to the effect of the exposure on the mediator (adiposity), while the natural direct effect captures the exposure-outcome effect that does not pass through the mediator. To interpret the effects causally several strong confounding assumptions must be met^18^: (1) control for exposure-outcome confounding; (2) control for mediator-outcome confounding; (3) control for exposure-mediator confounding; and (4) none of the mediator-outcome confounders are themselves affected by the exposure. While appropriate confounders will be applied, it is known that these assumptions are often violated in application and unmeasured confounding is likely present.^19^ We will evaluate the robustness of confounding assumptions through sensitivity analyses and in the discussion.

### Study population

This study uses anthropometric and caregiver reported data from the Millennium Cohort Study (MCS); a prospective birth cohort study of children born in the UK between 2000 and 2002. To ensure a representation of the total UK population, while also adequately sampling children typically underrepresented in surveys, the MCS employed stratified sampling at the electoral ward level with oversampling of disadvantaged and ethnic minority children.^20^ Technical details about the design, sampling outcomes and purpose of the MCS are available elsewhere.^20–22^ Table 1 shows the productive sample at each wave of the study.

**Table 1:** Details of the MCS sample, by wave of study^20 22^

Wave	Age*	Years conducted	Productive sample		Household response by wave (%)	Participated in all previous waves
			Children	Families		
1	9 months	2001–2002	19 517†	19 244‡	89.9	–
2	3 years	2004–2005	15 808	15 590	78.2	14 898
3	5 years	2006–2007	15 459	15 246	79.2	13 234
4	7 years	2008	14 043	13 857	81.1	11 721
5	11 years	2012–2013	13 469	13 287	81.4	10 448
6	14 years	2015	11 884	11 726	76.1	10 411

*Ages are approximate.

†Including 699 children recruited at wave 2.

‡Including 692 families recruited at wave 2.

MCS, Millennium Cohort Study.

**Figure 1:**
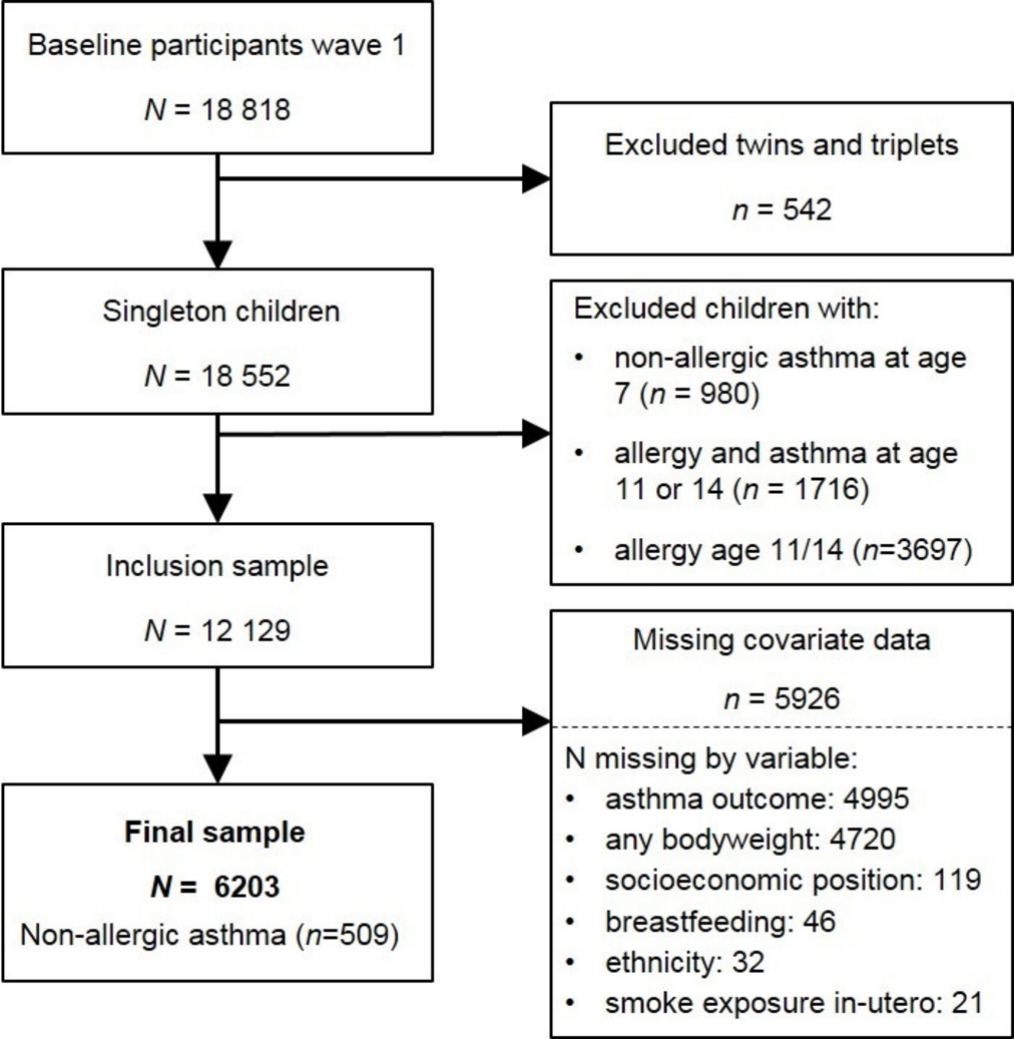
Flow chart of the sample selection.

The sample used in this current analysis is drawn from singleton cohort members who participated in the study at 9 months, 3, 7 and 11 or 14 years old; 59.1% of baseline singletons. Children with a history of non-allergic asthma at age 7 were excluded to ensure cases of non-allergic asthma in our study emerged after the study mediators, which were also recorded at age 7. Children with allergies or allergic asthma at age 11 or 14 were also excluded to ensure a valid control group was used.^23^ See figure 1 for a complete flow chart of sample selection. After excluding participants without complete data on all study variables, the final sample size was 6203. UK-level sampling weights were applied to the current analyses to adjust for sampling and attrition by wave 5.^24^ All data were retrieved from the UK Data Service.^25^

### Outcome measure

Asthma was defined by caregiver report of wheezing in the last 12 months and/or asthma ever, and allergy by caregiver report of hay fever and/or eczema ever. There is no objective measure of allergy in the MCS; however, these questions are drawn from the standardised and validated International Study of Asthma and Allergies in Childhood questionnaire.^26^ Those meeting the definition for asthma without allergies by age 11 or 14 (for those with no asthma or allergies at age 11) were defined as non-allergic asthmatics. The control group comprised children with no asthma or allergies at ages 11 and 14.

### Exposure

A polychoric principal components analysis was used on eight economic and social factors reported by parents at wave 1 (cohort member aged 9 months) to develop a composite index of socioeconomic position. One factor, self-rated financial status, was excluded due to low correlations with other items (<0.3), which led to a final index that accounted for 65.2% of the overall variance and had an eigenvalue of 4.56. The index comprises seven factors: age leaving full-time education, used to classify length of education (highest age used where parent responses differed); five-category National Vocational Qualification Classification, used to classify highest educational qualifications of parents; seven-category National Statistics Socio-economic Classification, used to rank current job type (highest classification used where parent responses differed); the Organisation for Economic Co-operation and Development below 60% poverty indicator, indicating households living in relative poverty; relationship status (married/cohabiting or other), to indicate family instability; housing tenure to define three categories of housing: own outright/mortgage, rent or other living circumstances (eg, live with parents, squat); and the number of rooms in the cohort member’s home (equivalised). The index was transformed into tertiles for analyses.

### Potential mediators

**Figure 2:**
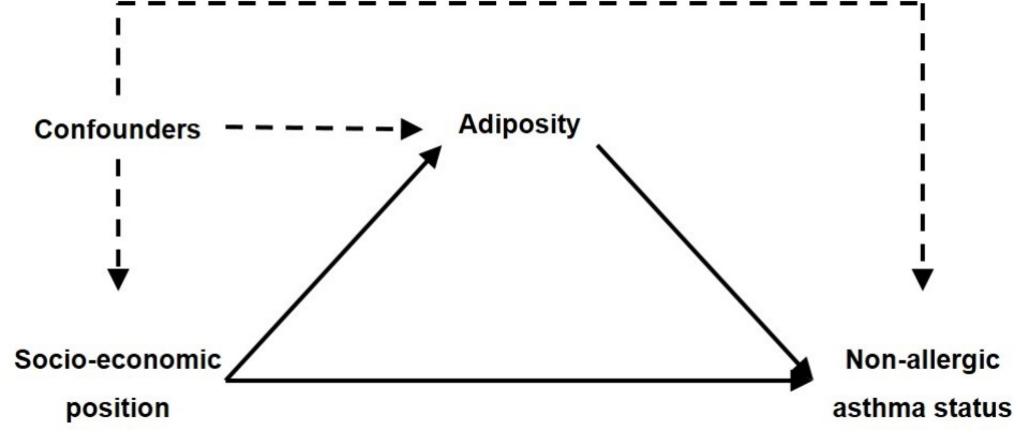
Hypothesised directed acyclic graph; adiposity measured in three separate models by body mass index (BMI) z-scores, waist-to-height ratio (WHtR) and waist circumference.

All body weight measurements were taken by trained interviewers in participants’ homes at age 7. BMI (kg/m²) was calculated by weight (kg), measured using Tanita scales, and height (m), measured using Leicester stadiometers. The BMI scores were then transformed into sex-age-specific z-scores using the UK90 growth references^27^ to create a new variable with a mean of 0 and SD of 1. Waist circumference (WC) was defined as the mean of two WC measurements (cm) taken using a tape measure. Waist-to-height ratio (WHtR) was calculated by mean WC (cm) divided by height (cm); expressed as one tenth of WHtR in results for interpretability. The three adiposity measurements were analysed as continuous variables to allow for potential quadratic effects on the outcome. Our hypothesised causal relationship between study variables is displayed in figure 2.

### Potential confounders

Confounders were selected on the basis of known associations with the exposure-mediator, mediator-outcome and/or exposure-outcome. All variables were reported by the child’s mother at wave 1 when the cohort member was aged 9 months: child’s sex (female/male); child’s ethnicity split into six categories (White, Mixed, Indian, Pakistani and Bangledeshi, Black or Black British, or other ethnic group); firstborn (yes/no); caesarean section delivery (yes/no); prenatal exposure to tobacco smoke (yes/no); postnatal exposure to tobacco (yes/no); and duration of breast feeding (<1 month, 1–3 months, >3 months).

### Analysis strategy

Stata V.14^28^ was used for all statistical analyses. Means (SDs) for continuous variables and percentages for other variables were used to describe the study sample and participants with missing data. Multivariable relationships between the exposure-mediators and exposure-outcome were explored using linear and logistic regression. Adiposity measures were tested in separate models due to multicollinearity, and were tested for non-linearity by adding a squared quadratic term (*y*=*a+bx+cx*^2^). Wald tests were used to assess linearity of variables, P<0.05.

To estimate the hypothesised mediation effect of adiposity we used Stata command ‘gformula’,^29^ a parametric g-computation procedure implemented using Monte Carlo simulations. gformula offers flexibility in comparison to traditional mediation methods, including modelling non-linearities and allowing for mediator-outcome confounding and exposure-mediator interaction. Three models, each with a separate measure of body weight, were run to estimate the natural direct and indirect effects in the relationship between socioeconomic position and non-allergic asthma. Results are presented as ORs with 95% CIs created from 1000 bootstrapped samples. gformula does not support svy weights; therefore, the final results were compared with weighted multivariable regressions. In the case of any significant mediation, Stata command ‘medsens’ was used to calculate how large the effect of an unmeasured mediator-outcome confounder needed to be to significantly alter the results.

## Results

Of the initial singleton cohort at wave 1, 51.1% children meeting our study inclusion criteria had complete data. Missingness in the outcome was biased towards lower socioeconomic position, male sex and non-white ethnicities, P<0.01. Characteristics of the final sample of 6203 children by socioeconomic group are viewable in table 2. Overall, there were slightly more women in the weighted sample than men (51.1%) and 86.4% of the cohort were white. At age 7, the average BMI was 0.33 SD higher than the UK90 reference standards, the average WC was 57.0 cm and the average WHtR was 0.46. Non-allergic asthma developed in 509 (8.5%) children of the final study sample between ages 7 and 14.

**Table 2:** Characteristics of the study population, by missing data and socioeconomic group

	Missing data sample*	Final sample†			
		Overall	Socioeconomic position		
			Low	Middle	High
Total unweighted (n)	5926	6203	1591	1987	2625
	**%**	**%**	**%**	**%**	**%**
Socioeconomic position					
Low	41.9	27.5	–	–	
Middle	34.5	32.6	–	–	
High	23.6	39.9	–	–	
Sex					
Female	45.2	50.9	50.6	51.7	51.0
Ethnic group					
White		78.2	86.4	77.1	88.2
Mixed	2.7	2.7	4.5	1.6	2.3
Indian	2.1	1.9	1.2	2.3	2.1
Pakistani and Bangladeshi	8.4	5.0	10.1	5.0	1.5
Black or Black British	6.0	2.7	5.0	1.8	1.7
Other	2.7	1.3	2.0	1.2	0.9
Firstborn					
Yes	43.7	50.7	45.6	52.8	52.2
Caesarean birth					
Yes	16.5	19.6	14.2	20.0	22.7
Tobacco exposure in utero					
Yes	21.5	16.1	30.0	17.0	5.8
Tobacco exposure in infancy					
Yes	21.4	14.9	30.2	15.3	4.1
Breast fed (months)					
<1	69.7	53.0	74.7	58.20	33.6
1–3	9.4	14.4	10.1	14.0	17.6
>3	21.0	32.7	15.2	27.8	48.8
Non-allergic asthma					
Yes	14.5	8.4	11.0	8.7	6.7
	**Mean (SD)**	**Mean (SD)**	**Mean (SD)**	**Mean (SD)**	**Mean (SD)**
Adiposity measures					
BMI z-score	0.46 (1.15)	0.33 (1.10)	0.36 (1.22)	0.40 (1.09)	0.27 (1.02)
Waist	57.5 (6.4)	57.0 (5.8)	57.4 (6.6)	57.2 (6.0)	56.7 (5.1)
WHtR	0.46 (0.04)	0.46 (0.04)	0.47 (0.04)	0.46 (0.04)	0.46 (0.04)

*Unweighted proportions and mean (SD) presented.

†Weighted proportions and mean (SD) presented.

BMI, body mass index; WHtR, waist-to-height ratio.

Results of multivariable exposure-outcome analyses are presented in table 3. Socioeconomic position was significantly associated with the development of non-allergic asthma after adjustment for confounders. Children in the lowest and middle socioeconomic groups had 77% (OR 1.77, 95% CI 1.30 to 2.40) and 32% (OR 1.32, 95% CI 1.01 to 1.70) increased odds of developing the outcome, respectively, compared with those in the highest group. All body weight measures had a significant non-linear association with non-allergic asthma, P<0.05, with peak odds at the highest end of the distribution for WHtR and WC and the lowest for BMI z-scores (see figure A1–3 in supplementary file 1). There was a significant linear relationship between socioeconomic position and adiposity measures. After adjustment for ethnicity, moving from a higher to lower socioeconomic group was associated with: 0.06 increased BMI z-score (β coefficient 0.06, 95% CI 0.01 to 0.11); 0.36 cm increase in WC (β coefficient 0.36, 95% CI 0.11 to 0.62); and a 0.6% increase in WHtR (β coefficient 0.06, 95% CI 0.04 to 0.07).

10.1136/jech-2017-209722.supp1 Supplementary file 1

**Table 3:** Weighted multivariable associations between socioeconomic position and non-allergic asthma

Adjustment	Socioeconomic position	OR	(95% CI)	P trend
Confounders*				
	High	1.00	(Baseline)	<0.001
	Middle	1.32	(1.01 to 1.70)	
	Low	1.77	(1.30 to 2.40)	
Confounders*+BMI z-score+BMI z-score²				
	High	1.00	(Baseline)	<0.001
	Middle	1.28	(0.99 to 1.66)	
	Low	1.72	(1.27 to 2.33)	
	BMI	1.00	(0.91 to 1.10)	<0.05
	+BMI²	1.05	(1.01 to 1.10)	
Confounders*+WC+WC^2^				
	High	1.00	(Baseline)	<0.001
	Middle	1.32	(1.02 to 1.71)	
	Low	1.76	(1.29 to 2.40)	
	WC	0.80	(0.65 to 0.98)	<0.05
	+WC²	1.00	(1.00 to 1.00)	
Confounders*+WHtR†+ WHtR²†				
	High	1.00	(Baseline)	<0.001
	Middle	1.33	(1.02 to 1.73)	
	Low	1.77	(1.30 to 2.40)	
	WHtR	0.04	(0.00 to 0.57)	<0.05
	+WHtR²	1.39	(1.08 to 1.80)	

*Confounders are sex, ethnicity, caesarean, firstborn, smoke in utero, smoke infancy and breast feeding.

†WHtR values presented as 1/10 WHtR.

BMI, body mass index; WC, waist circumference; WHtR, waist-to-height ratio.

Table 4 presents the natural direct and indirect effects of socioeconomic position on non-allergic asthma. The relationship between socioeconomic position and non-allergic asthma was not explained by BMI or WHtR. A slight negative indirect effect of WC was present; however, this was considered unsubstantial as it accounted for only 0.4% of the total effect. Significant associations were present in the natural direct effects of high compared with low socioeconomic groups and, to a lesser extent, high compared with middle groups. Overall, with WHtR included as a mediator, children in the lowest socioeconomic group had a 46% increased odds (OR 1.46, 95% CI 1.29 to 1.64) and children in the middle group had a 20% increased odds (OR 1.20, 95% CI 1.01 to 1.44) of developing non-allergic asthma compared with children in the highest group. These results align to the findings of weighted multivariable analyses (table 3). No interaction between socioeconomic position and mediators was found, P>0.05. As no mediation effect was found, sensitivity analyses were not conducted.

**Table 4:** Pathway from SEP to non-allergic asthma

	Low versus high socioeconomic position			Middle versus high socioeconomic position		
	OR*	(95% CI)	P	OR*	(95% CI)	P
BMI z-score						
NDE	1.433	(1.380 to 1.488)	<0.001	1.204	(0.883 to 1.641)	0.241
NIE	1.001	(0.996 to 1.007)	0.635	1.000	(0.999 to 1.001)	0.659
WHtR†						
NDE	1.455	(1.288 to 1.643)	<0.001	1.203	(1.005 to 1.441)	0.045
NIE	0.991	(0.978 to 1.004)	0.183	1.000	(0.997 to 1.003)	0.996
WC						
NDE	1.456	(0.866 to 2.449)	0.156	1.204	(0.970 to 1.495)	0.093
NIE	0.996	(0.992 to 1.000)	0.045	0.998	(0.995 to 1.001)	0.135

*Adjusted for sex, ethnicity, caesarean, firstborn, smoke in utero, smoke infancy and breast feeding.

†WHtR values presented as WHtR/10.

BMI, body mass index; NDE, natural direct effect; NIE, natural indirect effect; WC, waist circumference; WHtR, waist-to-height ratio.

## Discussion

Using a large UK prospective birth cohort, this study found no evidence to suggest that body weight mediates the effect of socioeconomic position on non-allergic asthma development in children between 7 and 14 years of age. In multivariable analyses, socioeconomic position and high or low adiposity were significant but independent risk factors for non-allergic asthma.

This research adds to the increasing literature that links disadvantage with the emergence of non-allergic asthma.^5 7 13^ An inverse association was found between socioeconomic position and non-allergic asthma, emphasising a social gradient to this health outcome. Prominently, this gradient persisted after adjustment for confounders. Previous research has found that higher rates of breast feeding and lower rates of maternal smoking during pregnancy account for the protective effect of high socioeconomic position^7 30^; however, the effect of socioeconomic position held after adjustment for these factors in our study. Our findings may differ due to our composite measure of deprivation used, which encompasses more factors than the single measure of parental education used in previous research.^5 7^ These results suggest that improving the socioeconomic welfare of children and promoting healthy weight might both be important to reduce the development of non-allergic asthma in mid-childhood.

High and low body weights at age 7 were related to an increased risk of non-allergic asthma. Previous prospective studies have found an association of body weight with non-allergic asthma in children with high BMI (obesity).^31 32^ Overweight and BMI as a continuous measure have had mixed results in other research, with some finding an effect in one gender only.^15 16 31^ BMI has been found to be a useful marker in adolescents and adults, but not in samples including only preadolescent children,^8 15 32^ likely due to changes in body mass that naturally occur in young children.^8^ The current study also found that extreme WHtR, and to a lesser extent WC, either high or low, had a significant independent association with non-allergic asthma. An association between central obesity and asthma has been previously observed in adult samples^14 33 34^; however, these studies failed to consider a non-linear relationship between the two conditions. Using a bioimpedance technique, Yiallouros *et al*^15^ found a U-shaped association between body fat percentage and asthma in preadolescent children. Our results suggest that using WHtR as a screening tool may be a simpler way to assess risk of non-allergic asthma in young populations.^35^

Employing g-computational techniques allowed us to model non-linear effects on a ratio scale in causal mediation analysis; however, strong assumptions accompany this method.^36^ Our results assume no further common causes of adiposity and non-allergic asthma apart from the confounders included. Indicators of early growth, for example, birth weight and gestational age, were not included although considered as confounders elsewhere.^37^ We did not account for these because our study focuses on the implications of weight status at age 7, and the addition of earlier growth variables would have likely changed the interpretation of our results. In addition, since there was no substantial mediating effect of adiposity in our analyses, it is unlikely that additional confounders would change the results. We need also to consider potential bias due to misclassification of the mediator. BMI, WHtR and WC are proxies of body fat, and do not distinguish as well between fat and lean mass compared with objective measures such as dual energy X-ray absorptiometry and bioimpedance analysers.^15 38^ However, a strength of our study is that more than one measure of bodyweight was tested, and these measures are quick and easy to administer in various settings. In addition, BMI scores were standardised by age and sex and similar results were found across all three bodyweight measures.

There are limitations to this study inherent to its design. Face-to-face interviews, used extensively in the MCS, are particularly prone to coverage and non-response biases.^39^ Socially patterned missingness was apparent in our data, which may have diluted the association between socioeconomic position, covariates and non-allergic asthma. The vast majority of missing data (97.1%) were due to non-response in waves 4 and 5. This pattern of attrition has been noted previously within the MCS and non-response weights have been designed to counter differential bias,^24^ and were applied in this study. In many instances, subjective methods of self-reporting or proxy-reporting were used to capture information. Detection of non-allergic asthma in particular relied on maternal reports rather than a clinical diagnosis or an objective measure, likely adding some unmeasurable misclassification bias to our outcome measure.^5^ Objective measures, such as a skin prick test for atopy, would be more a reliable measure in this instance.^17^ In addition, self-reporting of smoking in pregnancy is known to lead to underestimation.^40^ Nonetheless, the methods employed meant that a multitude of risk factors were available on a large and contemporary longitudinal sample. In total, non-allergic asthma was identified in 7.0% of the productive singleton cohort at age 7; a similar proportion to estimates using objective measures in previous research.^4^

This research shows that socioeconomic disadvantage and adiposity are both important, yet independent, risk factors for non-allergic asthma. The findings highlight the multidimensional approach needed to disrupt the trajectory leading to this respiratory condition. While the need for population-wide interventions to tackle childhood obesity in the UK, such as the National Child Measurement Programme,^41^ is not disputed, broader and earlier action to tackle social inequity is an unavoidable prerequisite to alleviating the burden of this chronic condition. In addition, this research adds to the literature showing that additional or alternative measures of bodyweight, aside from BMI, are useful when monitoring adiposity.^34 42^ High and low WHtR, in particular, should be measured when considering risk of future non-allergic asthma in paediatric populations.^15^

**What is already known on this subject**

- Socioeconomic position and adiposity both relate to non-allergic asthma in childhood.
- Whether adiposity accounts for socioeconomic differences in childhood asthma is unknown.

**What this study adds**

- Using prospective cohort data, we found that bodyweight did not alter the effect of socioeconomic position on subsequent non-allergic asthma in childhood.
- The results suggest that improving socioeconomic conditions and promoting healthy weight are both important in reducing the development of non-allergic asthma in early to mid-childhood.
