# Supplementary material for: Does adiposity mediate the relationship between socioeconomic position and non-allergic asthma in childhood?
Source: J Epidemiol Community Health. 2018 Jan 24;72(5):390–6. doi: 10.1136/jech-2017-209722 (PMC5909742; doi:10.1136/jech-2017-209722)
Supplement: Supplementary file 1 [file jech-2017-209722supp001.pdf]

Supplementary file 1 – Marginplots

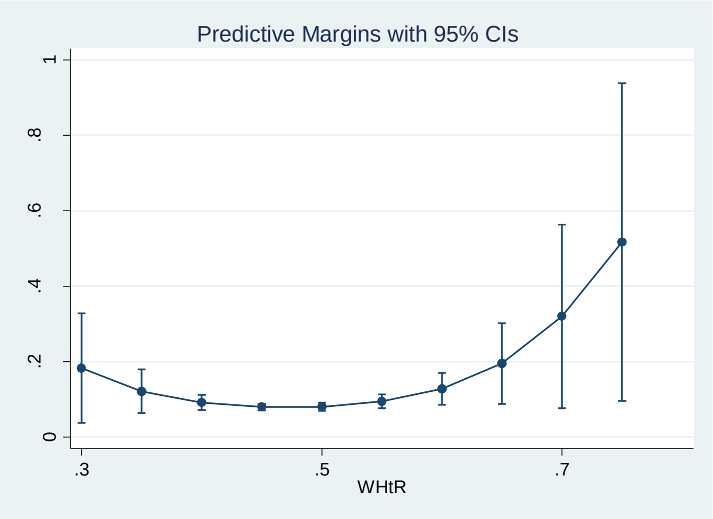

Figure A1. Adjusted predictions of WhtR on non-allergic asthma with 95% CI

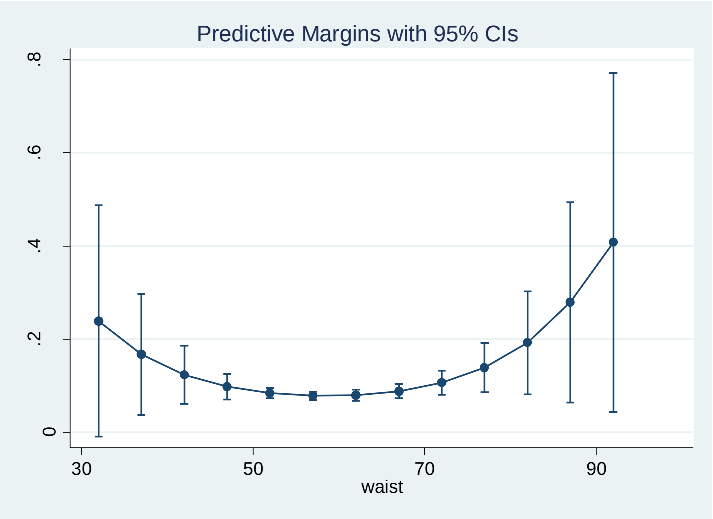

Figure A2. Adjusted predictions of WC on non-allergic asthma with 95% CI

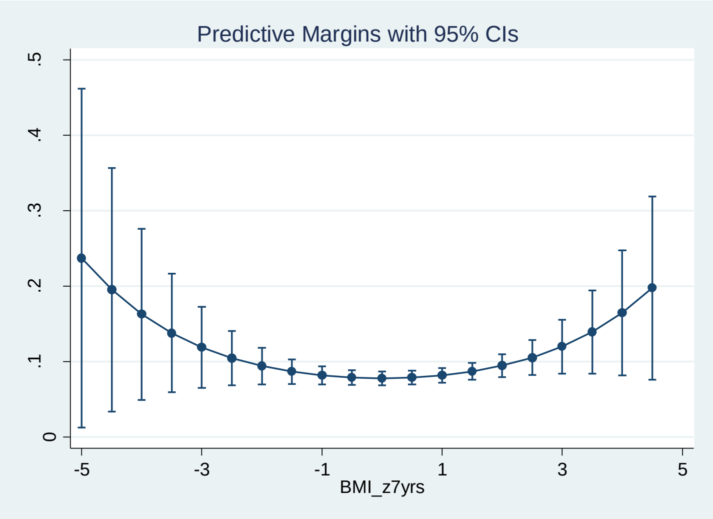

Figure A3. Adjusted predictions of BMI z-score on allergic-asthma with 95% CI
